# Gallium-Protoporphyrin IX Inhibits *Pseudomonas aeruginosa* Growth by Targeting Cytochromes

**DOI:** 10.3389/fcimb.2017.00012

**Published:** 2017-01-26

**Authors:** Sarah Hijazi, Paolo Visca, Emanuela Frangipani

**Affiliations:** Department of Science, Roma Tre UniversityRome, Italy

**Keywords:** aerobic respiration, antibacterial, cystic fibrosis, gallium, heme, infection, iron-uptake, terminal oxidases

## Abstract

*Pseudomonas aeruginosa* is a challenging pathogen due to both innate and acquired resistance to antibiotics. It is capable of causing a variety of infections, including chronic lung infection in cystic fibrosis (CF) patients. Given the importance of iron in bacterial physiology and pathogenicity, iron-uptake and metabolism have become attractive targets for the development of new antibacterial compounds. *P. aeruginosa* can acquire iron from a variety of sources to fulfill its nutritional requirements both in the environment and in the infected host. The adaptation of *P. aeruginosa* to heme iron acquisition in the CF lung makes heme utilization pathways a promising target for the development of new anti-*Pseudomonas* drugs. Gallium [Ga(III)] is an iron mimetic metal which inhibits *P. aeruginosa* growth by interfering with iron-dependent metabolism. The Ga(III) complex of the heme precursor protoporphyrin IX (GaPPIX) showed enhanced antibacterial activity against several bacterial species, although no inhibitory effect has been reported on *P. aeruginosa*. Here, we demonstrate that GaPPIX is indeed capable of inhibiting the growth of clinical *P. aeruginosa* strains under iron-deplete conditions, as those encountered by bacteria during infection, and that GaPPIX inhibition is reversed by iron. Using *P. aeruginosa* PAO1 as model organism, we show that GaPPIX enters cells through both the heme-uptake systems *has* and *phu*, primarily *via* the PhuR receptor which plays a crucial role in *P. aeruginosa* adaptation to the CF lung. We also demonstrate that intracellular GaPPIX inhibits the aerobic growth of *P. aeruginosa* by targeting cytochromes, thus interfering with cellular respiration.

## Introduction

**Figure 1:**
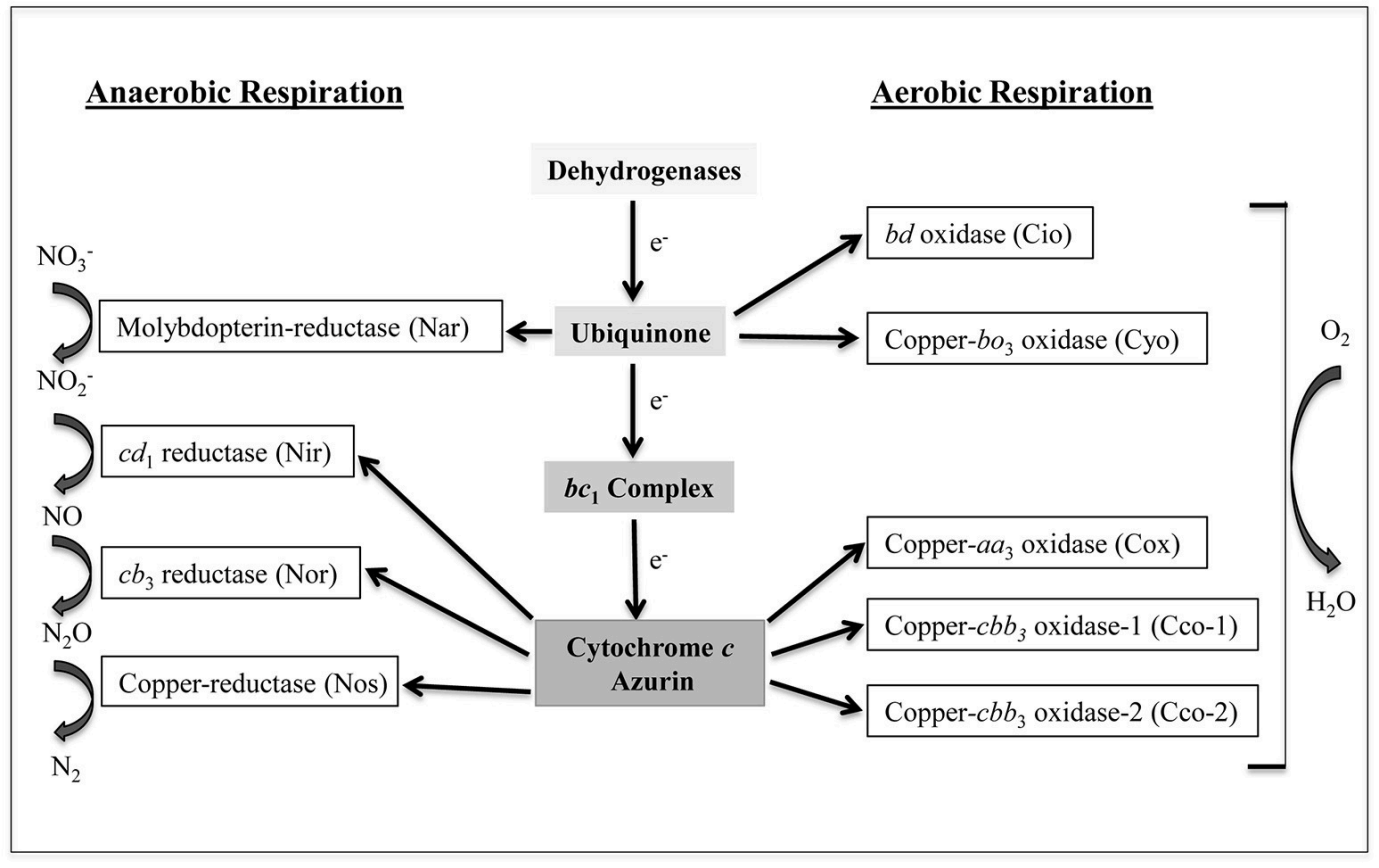
**Branched respiratory chain of ***P. aeruginosa***. Cio, Cyo, Cox, Cco-1, and Cco-2 represent the five terminal oxidases that reduce oxygen to water under aerobic conditions**. Cio and Cyo are quinol oxidases while Cox, Cco-1, and Cco-2 are cytochrome *c* oxidases. Nar, Nir, Nor, and Nos are nitrate reductase, nitrite reductase, nitric oxide reductase, and nitrous oxide reductase, respectively. These enzymes transfer electron to nitrogen oxides under anaerobic conditions. Nar receives electrons directly from the quinone pool while the other three receive electrons *via* the cytochrome *c* or from the small blue-copper protein azurin. *a, b, c*, and *d* represent different types of low-spin heme while *a*_3_, *b*_3_, *d*_1_, and *o*_3_ indicate the high-spin ones (modified from Arai, 2011).

*Pseudomonas aeruginosa* is a challenging bacterial pathogen due to both innate and acquired resistance to several antibiotics (Moore and Flaws, 2011). This bacterium is capable of causing a variety of infections, including chronic lung infection, which represents the main cause of morbidity and mortality in patients suffering from cystic fibrosis (CF) (Murphy, 2006; Davies et al., 2007). The success of *P. aeruginosa* as an opportunistic pathogen relies, at least in part, on its metabolic versatility, including the ability to obtain energy from different sources under a variety of environmental conditions (Williams et al., 2007; Arai, 2011). *P. aeruginosa* possesses a branched respiratory chain terminated by oxygen or nitrogen oxides, to allow growth by aerobic respiration or by denitrification under anaerobic conditions, respectively (reviewed in Arai, 2011). Moreover, *P. aeruginosa* is able to ferment arginine and pyruvate anaerobically (Vander et al., 1984; Eschbach et al., 2004). Aerobic respiration in *P. aeruginosa* relies on five terminal oxidases (Matsushita et al., 1982, 1983; Fujiwara et al., 1992; Cunningham and Williams, 1995; Cunningham et al., 1997; Stover et al., 2000; Comolli and Donohue, 2002, 2004). Three of these enzymes, the *aa*_3_ terminal oxidase (Cox), the *cbb*_3_-1 (Cco-1), and the *cbb*_3_-2 (Cco-2) are cytochrome *c*-type oxidases, while the other two, i.e., the cyanide-insensitive oxidase (Cio) and the *bo*_3_ oxidase (Cyo), are quinol oxidases (Figure 1). All these terminal oxidases contain heme, and are differentially expressed depending on the growth conditions, likely as a consequence to their different affinity for oxygen (Alvarez-Ortega and Harwood, 2007; Kawakami et al., 2010). Denitrification is ensured by a set of enzymes which sequentially convert nitrate (${\text{NO}}_{3}^{-}$) to molecular nitrogen (N_2_). Among the denitrification enzymes, only nitrite reductase (Nir) and nitric oxide reductase (Nor) contain heme as a cofactor (Figure 1).

Like almost all pathogenic bacteria, *P. aeruginosa* has an absolute need for iron to cause infections and to persist within the host (Ratledge and Dover, 2000). Iron is required as a cofactor of many key enzymes involved in respiration, DNA synthesis and defense against reactive oxygen species (Andrews et al., 2003). However, in the human host, iron is poorly available to bacteria due to its incorporation into heme-containing molecules (e.g., hemoglobin and myoglobin) and iron carrier proteins (e.g., transferrin and lactoferrin) (Weinberg, 2009). This iron-withholding capacity represents the first line of the host defense against invading pathogens, a phenomenon known as “nutritional immunity” (Skaar, 2010). To circumvent iron-limitation, *P. aeruginosa* possesses several systems that actively acquire this essential metal, such as (i) the production of the siderophores pyoverdine (Pvd, Meyer and Abdallah, 1978; Cox and Adams, 1985) and pyochelin (Pch, Cox et al., 1981; Heinrichs et al., 1991); (ii) the ability to utilize a wide range of siderophores synthesized by other organisms (Cornelis and Matthijs, 2002; Cornelis et al., 2009); (iii) the ability to acquire Fe(II) through the Feo system (Cartron et al., 2006). In addition, *P. aeruginosa* can utilize heme-iron, by expressing two distinct heme-uptake systems, namely *phu* and *has* (Ochsner et al., 2000). The *phu* system allows the direct acquisition of heme from hemoproteins, which bind to the outer membrane receptor PhuR (Ochsner et al., 2000). In the *has* system a secreted hemophore HasA withdraws heme from hemoproteins and delivers it to the outer membrane receptor HasR (Létoffé et al., 1998). Given the similarity with the well-known *has* system of *Serratia marcescens* (Rossi et al., 2003; Létoffé et al., 2004), it is likely that the *has* system of *P. aeruginosa* positively regulates its own expression, *via* the sigma factor HasI and anti-sigma HasS, upon interaction of heme-loaded HasA with the HasR receptor (Llamas et al., 2014). The expression of both *has* and *phu* heme-uptake systems is shut down in the presence of sufficient intracellular iron, due to the negative regulation exerted by the ferric-uptake regulator (Fur) protein (Ochsner et al., 2000).

It has been shown that *P. aeruginosa* aerobic respiration and iron-uptake capabilities play pivotal roles during chronic lung infection in CF patients. In particular, three terminal oxidases (Cco-1, Cco-2, and Cio) sustain bacterial growth in the CF lung, a particular environment where *P. aeruginosa* iron-uptake abilities are sought to evolve toward heme utilization (Alvarez-Ortega and Harwood, 2007; Marvig et al., 2014; Nguyen et al., 2014).

The paucity of effective antibiotics to treat *P. aeruginosa* infections have made bacterial respiration and/or iron metabolism promising targets for the development of new anti-*Pseudomonas* drugs (Ballouche et al., 2009; Foley and Simeonov, 2012; Imperi et al., 2013). The possibility of using iron mimetics as novel therapeutics to interfere with iron metabolism has been exploited (Kaneko et al., 2007; Banin et al., 2008; Minandri et al., 2014). Ga(NO_3_)_3_, the active component of the FDA-approved formulation Ganite®, has successfully been repurposed as an antimicrobial drug (Bonchi et al., 2014; Rangel-Vega et al., 2015). Interestingly, Ga(NO_3_)_3_ has been shown to be very active against *P. aeruginosa*, by interfering with iron-dependent metabolic pathways (Kaneko et al., 2007; Bonchi et al., 2015). The antibacterial proprieties of Ga(III) reside in the fact that, different from Fe(III), Ga(III) cannot be reduced under physiological conditions. However, redox cycling is critical for many of iron-dependent biological functions, including respiration (Breidenstein et al., 2011). Moreover, the heme-mimetic GaPPIX [i.e., Ga(III) coupled with the heme precursor protoporphyrin IX] has been shown to possess a good antibacterial activity against several bacterial species, including *Staphylococcus aureus* and *Acinetobacter baumannii* (Stojiljkovic et al., 1999; Arivett et al., 2015; Chang et al., 2016). GaPPIX is likely to exploit heme-uptake routes to enter bacterial cells, where it could substitute for heme in heme-containing enzymes, including cytochromes, catalases, and peroxidases, resulting in the perturbation of vital cellular functions (Stojiljkovic et al., 1999). Due to the similarity between GaPPIX and heme, GaPPIX is predicted to interfere with heme-dependent *b*-type cytochromes, thus impairing their function and ultimately inhibiting bacterial respiration.

In this work, the *in vitro* effect of GaPPIX on *P. aeruginosa* was tested under iron-depleted conditions, as those encountered during infection. The entrance routes of GaPPIX into *P. aeruginosa* cells and possible targets of GaPPIX were investigated. We demonstrate that the sensitivity of *P. aeruginosa* to GaPPIX depends on both intracellular iron levels and the expression of heme-uptake systems. Furthermore, we show that GaPPIX enters *P. aeruginosa* cells mainly through the heme-uptake receptor PhuR. Evidence is also provided that intracellular GaPPIX inhibits the aerobic growth of *P. aeruginosa* by targeting heme-dependent *b*-type cytochromes.

## Materials and methods

### Bacterial strains and growth conditions

Strains and plasmids used in this work are listed in Table 1. *P. aeruginosa* clinical isolates are listed in Table S1. *P. aeruginosa* strains from frozen cultures were maintained on Luria Bertani (LB) agar before being transferred to liquid culture media. Bacteria were cultured in iron-free Casamino Acids medium (DCAA, Visca et al., 1993) supplemented or not with 100 μM of FeCl_3_ at 37°C, with vigorous shaking. When required, antibiotics were added to the media at the following concentrations for *Escherichia coli*, with the concentrations used for *P. aeruginosa* shown in parentheses: Ampicillin 100 μg/ml; carbenicillin (300 μg/ml in LB and 200 μg/ml in DCAA); and tetracycline 12.5 μg/ml (100 μg/ml). DCAA agar plates were prepared by the addition of 15 g/l bacteriological agar (Acumedia, Neogen corporation). When GaPPIX was required, a 50 mM of stock solution of GaPPIX (Frontier Scientific) was prepared in dimethyl sulfoxide (DMSO) and stored at 4°C in the dark. When Ga(NO_3_)_3_ was required, a 100 mM of stock solution of Ga(NO_3_)_3_ (Sigma-Aldrich), was prepared in double-distilled water and stored at −20°C.

**Table 1:** **Bacterial strains and plasmids used in this study**.

**Strain or plasmid**	**Genotype and/or relevant characteristics**	**Reference or source**
**STRAINS**		
***P. aeruginosa***		
PAO1	ATCC15692 (wild type, prototroph)	American type culture collection
Δ*hasR*	PAO1Δ*hasR*	This work
Δ*phuR*	PAO1Δ*phuR*	This work
*ΔhasR*Δ*phuR*	PAO1Δ*hasR*Δ*phuR*	Minandri et al., 2016
Δ*pvdA*	PAO1Δ*pvdA*	Imperi et al., 2008
Δ*pchD*	PAO1Δ*pchD*	Frangipani et al., 2014
Δ*pvdA*Δ*pchD*	PAO1Δ*pvdA*Δ*pchD*	Visca et al., 2013
Δ*cio*	PAO1 containing a 2400-bp deletion in the *cioAB* locus	This work
Δ*cox*	PAO1 containing a 4109-bp deletion in the *coxBA*-PA0107*-coIII* locus	This work
Δ*cyo*	PAO1 containing a 4830-bp deletion in the *cyoABCDE* operon	This work
Δ*cco*	PAO1 containing a 6445-bp deletion in the two adjacent *ccoNOQP1* and *ccoNOQP2* operons	This work
Δ*cyo*Δ*cio*	PAO1 mutated in both *cyo* and *cio*	This work
Δ*cyo*Δ*cco*	PAO1 mutated in both *cyo* and *cco*-1,2	This work
Δ*cyo*Δ*cio*Δ*cox*	PAO1 mutated in *cyo, cio* and *cox*	This work
Δ*cyo*Δ*cco*Δ*cox*	PAO1 mutated in *cyo, cco* and *cox*	This work
***E. coli***		
DH5αF′	*recA1 endA1 hsdR17 supE44 thi-1 gyrA96 relA1* Δ(*lacZYA-argF*) U169 [Φ*80dlacZ*Δ*M15*] Nal^R^	Sambrook et al., 1989
S17-1 λpir	*recA, thi, pro, hsdR*-M+RP4: 2-Tc:Mu: Km Tn7 λpir, Tp^R^ Sm^R^	Simon et al., 1983
**PLASMIDS**		
pDM4	Suicide vector; *sacBR, oriR6K*, Cm^R^	Milton et al., 1996
pME7541	Suicide construct used for deletion of the *cioAB* operon; Tc^R^	Frangipani et al., 2008
pME9302	Suicide construct used for deletion of the *coxB-coII*I cluster; Tc^R^	Frangipani et al., 2008
pME9303	Suicide construct used for deletion of the *cyoABCDE* operon; Tc^R^	Frangipani et al., 2008
pME9308	Suicide construct used for deletion of the two adjacent *ccoNOQP* operons; Tc^R^	Frangipani et al., 2008
pUCP18	*E. coli-Pseudomonas* shuttle vector derived from pUC18; ColE1, pRO1600, Ap^R^, Cb^R^	Schweizer, 1991
pUCP*hasR*	pUCP18 derivative carrying the coding sequence of *hasR* with its own promoter	This study
pUCP*phuR*	pUCP18 derivative carrying the coding sequence of *phuR* with its own promoter	This study
pUCP*phuRhasR*	pUCP18 derivative carrying the coding sequence of *phuR* and *hasR* with their own promoters	Minandri et al., 2016
pDM4Δ*hasR*	pDM4 derivative carrying the flanking regions of the *hasR* coding sequence	Minandri et al., 2016
pDM4Δ*phuR*	pDM4 derivative carrying the flanking regions of the *phuR* coding sequence	Minandri et al., 2016
**Oligonucleotides**	**Sequence 5′-3′**	**Restriction site**
*hasR* compl FW	CGGGGTACCGGCGGGAGTGACGCTGC	KpnI
*hasR* compl RV	GAAGATCTCCTTCACTGGGCAAAACGG	BglII
*phuR* compl FW	CCGGAATTCGAAAGGCTGGGAGTGCTG	EcoRI
*phuR* compl RV	CGGGGTACCACCTGTGGCATGGAAAGC	KpnI

*Tc^R^, tetracycline resistant; Ap^R^, ampicillin resistant; Cm^R^, chloramphenicol resistant; Cb^R^, carbenicillin resistant; restriction sites in the oligonucleotides are underlined*.

### Susceptibility testing

The activity of GaPPIX, Ga(NO_3_)_3_ and Hemin (Hm) (Sigma-Aldrich) on *P. aeruginosa* was tested in 96-well microtiter plates (Falcon). Briefly, bacterial cells were grown over-night in DCAA supplemented with 100 μM FeCl_3_ in order to obtain high cell densities, then washed in saline and diluted to an OD_600_ of 0.01 in 200 μl of DCAA containing increasing concentrations (0–100 μM) of GaPPIX, Ga(NO_3_)_3_ or Hm. Microtiter plates were incubated for 24 h at 37°C with gentle shaking (120 rpm). Growth (OD_600_) was measured in a Wallac 1420 Victor3 V multilabel plate reader (PerkinElmer). The minimum inhibitory concentration (MIC) of gallium compounds was visually determined as the lowest concentration that completely inhibited *P. aeruginosa* growth. As a control experiment the same procedure was performed, except that 100 μM FeCl_3_ was added in the medium containing the highest concentration of gallium compounds tested (100 μM).

The antibacterial activity of gallium compounds was also assessed by disk diffusion assays. Briefly, cells from an over-night culture in DCAA supplemented with 100 μM FeCl_3_ were washed and diluted in saline to OD_600_ = 0.1, then seeded on the surface of DCAA agar plates supplemented or not with FeCl_3_. Sterile 6-mm blank disks (ThermoFisher-Oxoid) soaked with 10 μl of a 15 mM solution of either GaPPIX or Ga(NO_3_)_3_ were deposited on the agar surface and the Zone Of growth Inhibition (ZOI) was measured (in mm) after 16 h of incubation at 37°C.

To observe the rescue effect of Hm and Hemoglobin (Hb), disks were soaked with 10 μl of a 7.5 mg/ml solution of bovine hemin chloride (Sigma-Aldrich) in 10 mM NaOH or bovine hemoglobin (Sigma-Aldrich) in phosphate buffered saline (PBS) and deposited on the plate surface nearby the disk soaked with GaPPIX. The appearance of a half-moon-shaped growth area around the disk soaked with Hm or Hb was detected after 16 h of incubation at 37°C.

### Construction of plasmids for the expression of heme receptors

Plasmid preparations and DNA cloning were performed according to standards methods (Sambrook et al., 1989). Restriction and DNA modifying enzymes were used following the instructions of the manufacturers. Oligonucleotide primers are listed in Table 1. To express *hasR* in the Δ*hasR*Δ*phuR* mutant, a 2932 bp fragment containing the *hasR* gene with its own promoter region was amplified by PCR from the PAO1 genome using primers *hasR* compl FW and *hasR* compl RV (Table 1). The product was then digested with KpnI and BglII and directionally cloned into the corresponding sites of the shuttle vector pUCP18, giving plasmid pUCP*hasR*. To express *phuR* in Δ*hasR*Δ*phuR* mutant, a 2575 bp fragment containing the *phuR* gene with its own promoter region was amplified by PCR from the PAO1 genome using primers *phuR* compl FW and *phuR* compl RV (Table 1). The product was then digested with EcoRI and KpnI and directionally cloned into the corresponding sites of the shuttle vector pUCP18, giving plasmid pUCP*phuR*. To express *hasR* and *phuR* in the Δ*hasR*Δ*phuR* mutant strain, the pUCP*hasRphuR* plasmid previously described (Minandri et al., 2016) was used.

### Generation of *P. aeruginosa* mutants

For mutant construction, *E. coli* and *P. aeruginosa* strains were grown in LB, with or without antibiotics, at 37 and 42°C, respectively, with vigorous aeration. Previously described suicide plasmids (Table 1) were used according to procedures detailed elsewhere (Milton et al., 1996; Frangipani et al., 2008).

### Measurement of cytochrome *c* oxidase activity in *P. aeruginosa* intact cells

Cytochrome *c* oxidase activity was assayed by using the artificial electron donor *N*,*N*,*N'*,*N'*tetramethyl-*p*-phenylene diamine (TMPD) (Fluka). Briefly, bacteria were grown over-night in DCAA supplemented with 100 μM FeCl_3_, then washed in saline and inoculated in DCAA to a final OD_600_ = 0.05. When the mid-exponential growth phase was reached (≈6 h post inoculum), cells were washed once in saline and adjusted to an OD_600_ = 1 (corresponding to ≈10^9^ CFU/ml).

Then, 10^8^ bacterial cells (100 μl) were suspended in 1.4 ml of 33 mM potassium phosphate buffer (KPi, pH 7.0). The reaction was started by the addition of 5 μl of a 0.54 M TMPD solution to the sample cuvette. The rate of TMPD oxidation was recorded spectrophotometrically at 520 nm for 8 min at 25°C. Results were expressed as μmol TMPD oxidized/min^−1^/10^8^cells using 6.1 as the millimolar extinction coefficient of TMPD (Matsushita et al., 1982).

### Isolation of outer membrane proteins (OMPs) and SDS-PAGE analysis

OMPs were isolated following the sarcosyl solubilization method (Filip et al., 1973), with some modifications. Briefly, bacteria from over-night cultures in DCAA supplemented with 100 μM FeCl_3_ and 200 μg/ml Cb were washed in saline, then diluted to OD_600_ = 0.05 in 60 ml DCAA supplemented with 200 μg/ml Cb, and incubated over-night at 37°C. Cells were collected by centrifugation (2500 × *g*, 20 min), washed with 5 ml of 30 mM Tris HCl (pH 8, Sigma-Aldrich) and suspended in 1 ml of the same buffer. Bacteria were lysed by sonication in an ice bath (8 × 20 s cycles in a Sonics Vibra-Cell™ VCX 130 sonicator), punctuated by 20 s intervals (50% power). Phenyl methyl sulfonyl fluoride (PMSF, Sigma-Aldrich) was added to cell lysate at 1 mM final concentration. Unbroken cells were removed by centrifugation at 2400 × *g* for 20 min, and supernatants were transferred to fresh tubes. Sarcosyl (N-laurylsarcosinate sodium salt, Sigma) was added to the supernatant to a final concentration of 2%. After 1 h incubation at room temperature with gentle shaking, the mixture was centrifuged for 2 h at 55,000 × *g* at 4°C. OMP pellets were suspended in 40 μl 2 x SDS-PAGE loading dye (Sambrook et al., 1989), boiled for 10 min, then separated by 8% SDS-PAGE and visualized by Coomassie brilliant blue staining.

### Statistical analysis

Statistical analysis was performed with the software GraphPad Instat (GraphPad Software, Inc., La Jolla, CA), using One-Way Analysis of Variance (ANOVA), followed by Tukey-Kramer Multiple Comparisons Test.

## Results

### *P. aeruginosa* is inhibited by GaPPIX under iron-deplete conditions

**Figure 2:**
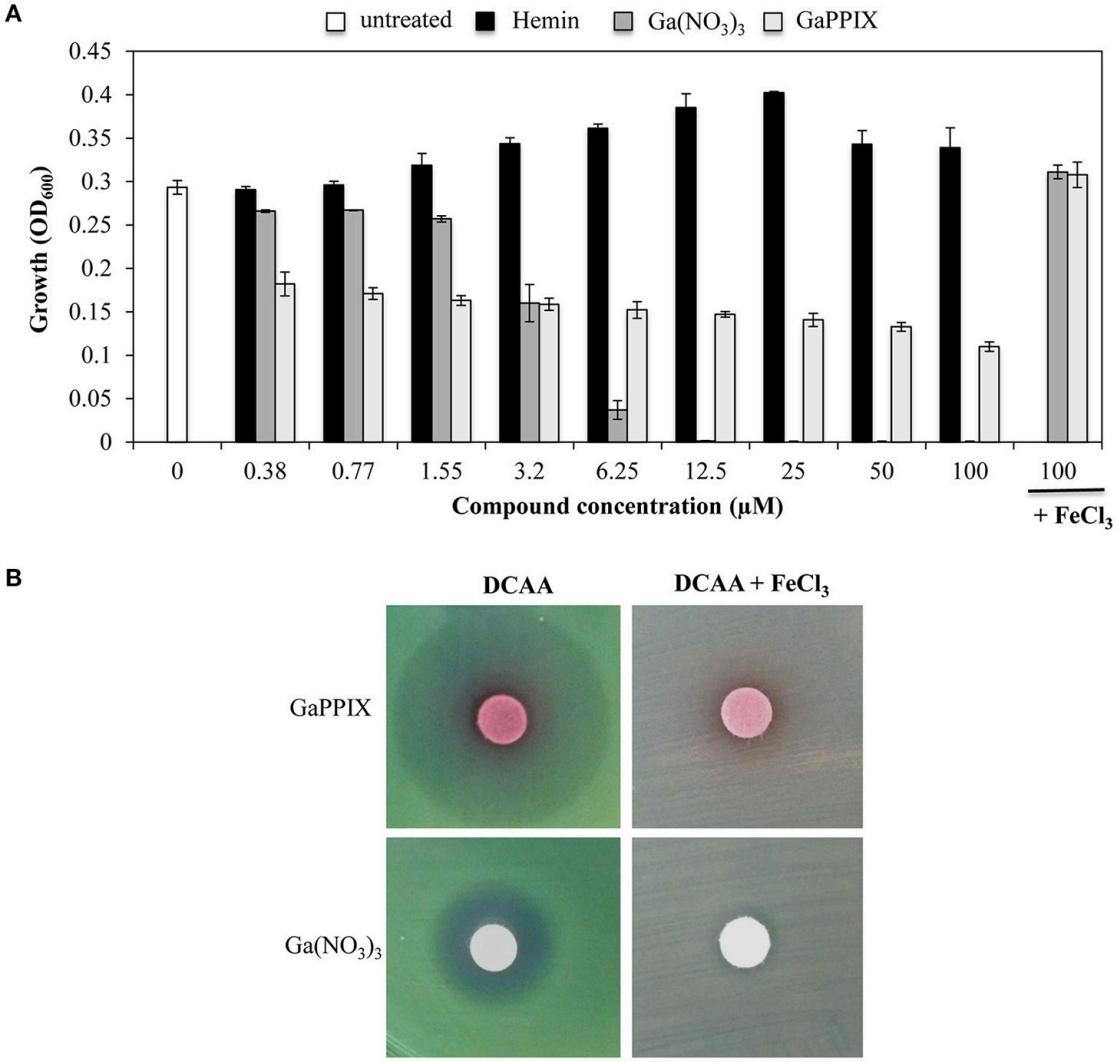
**GaPPIX inhibits ***P. aeruginosa*** PAO1 growth under iron-deplete conditions. (A)** *P. aeruginosa* PAO1 was grown for 24 h at 37°C in DCAA in the presence of different concentrations of Hemin (black bars), Ga(NO_3_)_3_ (dark gray bars), GaPPIX (light gray bars), or nothing (white bar). Control cultures were supplemented with 100 μM FeCl_3_ and 100 μM of either Ga(NO_3_)_3_ or GaPPIX. Values are the mean of 3 independent experiments, each one performed in duplicate ± the standard deviation. **(B)** Approximately 5 × 10^6^ *P. aeruginosa* PAO1 cells were seeded on the surface of DCAA agar plates, supplemented or not with 600 μM FeCl_3_, as indicated on top. Then, disks soaked with 10 μl of a 15 mM solution of either GaPPIX or Ga(NO_3_)_3_ were deposited on the agar surface, as indicated on the left. Plates were incubated for 16 h at 37°C. Images are representative of three independent experiments yielding similar results.

It has been previously reported that GaPPIX has no effect on *P. aeruginosa* (Stojiljkovic et al., 1999). This results is quite surprising given that *P. aeruginosa* is able to utilize heme as an iron source, by expressing two heme-uptake systems, i.e., *has* and *phu* (Ochsner et al., 2000). However, since the effect of GaPPIX has previously been investigated in iron-rich media (Stojiljkovic et al., 1999), we sought that under these conditions iron availability would have impaired Ga(III) activity. To verify this hypothesis, we preliminary tested the effect of GaPPIX on *P. aeruginosa* PAO1 growth using the iron-poor medium DCAA (Visca et al., 1993), supplemented with increasing concentrations of GaPPIX, the iron-binding porphyrin Hemin, or Ga(NO_3_)_3_, the latter resulting very active on *P. aeruginosa* in this medium (Bonchi et al., 2015). Ga(NO_3_)_3_ completely inhibited *P. aeruginosa* growth at 12.5 μM, and its activity was abrogated by the addition of FeCl_3_ (Figure 2A) consistent with previous findings (Kaneko et al., 2007; Frangipani et al., 2014). Although the minimal inhibitory concentration (MIC) could not be determined for up to 100 μM GaPPIX (Figure 2A), exposure of PAO1 to GaPPIX reduced bacterial growth by 50% (IC_50_) at 12.5 μM (Figure 2A). Also in the case of GaPPIX, growth inhibition was completely reversed by the addition of FeCl_3_ (Figure 2A). As expected, exposure *P. aeruginosa* PAO1 to Hemin promoted bacterial growth at concentrations ranging between 1.55 and 25 μM, in line with the ability of *P. aeruginosa* to use Hemin as an iron source (Ochsner et al., 2000).

The GaPPIX susceptibility of *P. aeruginosa* PAO1 was also tested using the disk diffusion assays in DCAA agar plates supplemented or not with an excess of FeCl_3_ (600 μM) (Figure 2B). In FeCl_3_-supplemented DCAA, both GaPPIX and Ga(NO_3_)_3_ caused no inhibition of PAO1 growth. Conversely, in DCAA a clear ZOI was observed around the GaPPIX and Ga(NO_3_)_3_ disks (Figure 2B). Different from the ZOI formed by Ga(NO_3_)_3_, the ZOI formed by GaPPIX was less transparent (Figure 2B), consistent with the evidence that no MIC (full inhibition) could be determined for GaPPIX in liquid DCAA (Figure 2A). Although more transparent, the ZOI caused by Ga(NO_3_)_3_ was smaller than that of GaPPIX (Figure 2B). These preliminary data indicate that iron-deplete conditions render *P. aeruginosa* PAO1 susceptible to GaPPIX-mediated growth inhibition.

### The response of *P. aeruginosa* cells to GaPPIX depends on intracellular iron carryover

**Figure 3:**
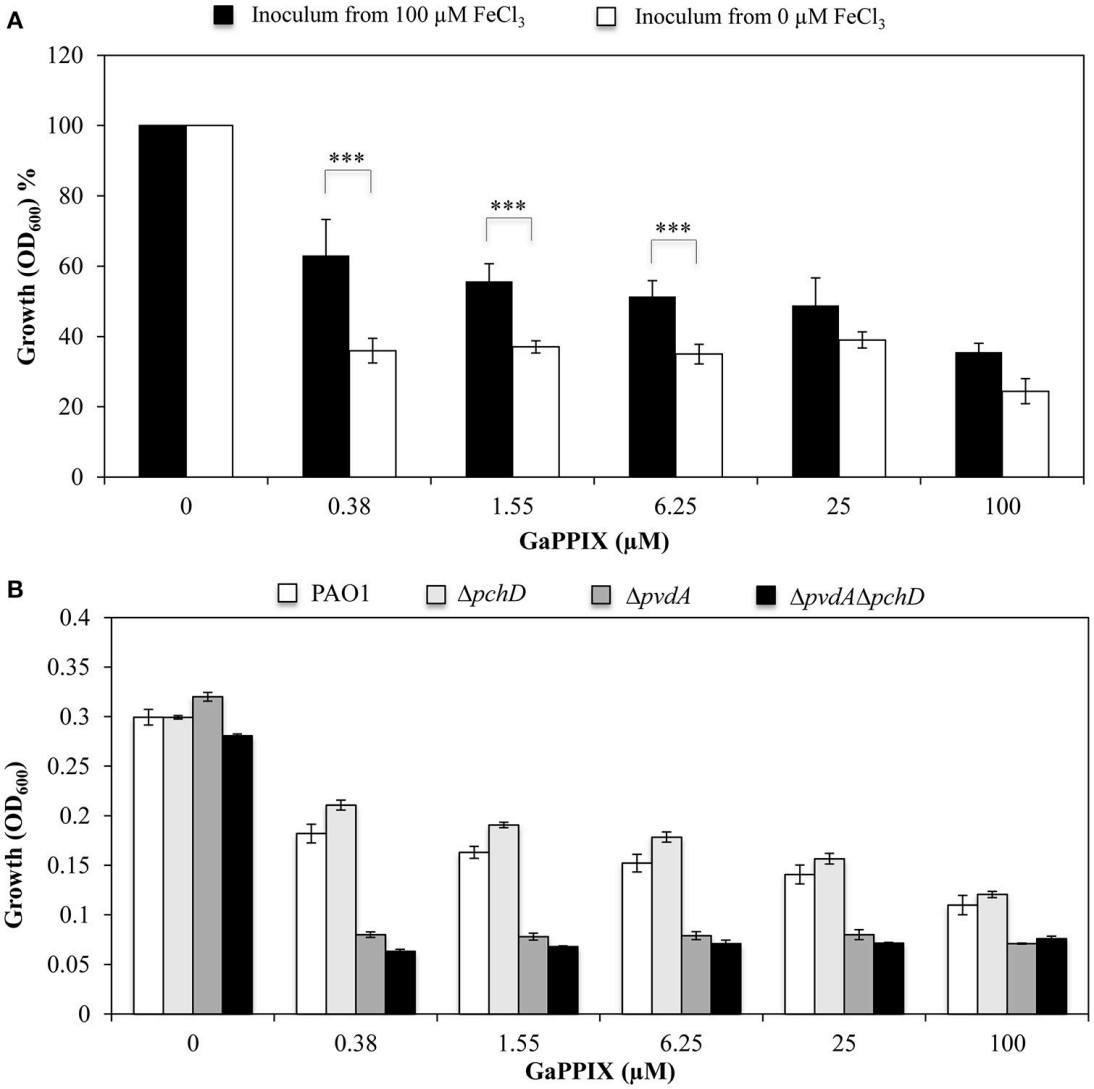
**The response of ***P. aeruginosa*** to GaPPIX depends on intracellular iron carryover. (A)** *P. aeruginosa* PAO1 cells from an inoculum with 100 μM FeCl_3_ (black bars) and without FeCl_3_ (white bars) were grown in DCAA supplemented with increasing concentrations of GaPPIX for 24 h at 37°C. The values are expressed as the percentage relative to the untreated cultures, and represent the mean of four independent experiments, each one performed at least in duplicate ± the standard deviation. Asterisks indicate statistically significant differences relative to a culture derived from cells grown in the presence of 100 μM FeCl_3_ (^***^P < 0.001). **(B)** *P. aeruginosa* PAO1 (white bars), Δ*pchD* (light gray bars), Δ*pvdA* (dark gray bars), and Δ*pvdA*Δ*pchD* (black bars) were grown in DCAA supplemented with increasing concentrations of GaPPIX for 24 h at 37°C. Values are the mean of two independent experiments, each one performed in duplicate ± the standard deviation.

The above results prompted us to investigate the effect of the intracellular iron content on GaPPIX-dependent growth inhibition. To this aim, the effect of GaPPIX was compared between *P. aeruginosa* PAO1 cells that had been pre-cultured in either DCAA containing 100 μM FeCl_3_ (to increase the intracellular iron content) or DCAA without FeCl_3_ (to lower the intracellular iron content). Iron-starved bacterial cells were significantly more susceptible to GaPPIX (*P* < 0.001) compared with those pre-cultured with FeCl_3_ (Figure 3A). In particular, upon the addition of 0.38 μM GaPPIX, the growth of iron-starved PAO1 cells was reduced by 40% compared with cells pre-cultured in the presence of 100 μM FeCl_3_ (Figure 3A).

To further investigate the correlation between the intracellular iron content and GaPPIX-dependent growth inhibition, GaPPIX susceptibility was evaluated on *P. aeruginosa* mutants impaired in Fe(III)-siderophore uptake systems, i.e., mutants unable to synthesize pyoverdine (Δ*pvdA*), pyochelin (Δ*pchD*), or both siderophores (Δ*pvdA*Δ*pchD*) (Figure 3B). While GaPPIX-dependent growth inhibition was similar in the wild type and the Δ*pchD* mutant, both Δ*pvdA* and Δ*pvdA*Δ*pchD* mutants were extremely sensitive to GaPPIX (Figure 3B). In particular, 0.38 μM GaPPIX inhibited the growth of the Δ*pvdA* and Δ*pvdA*Δ*pchD* mutant strains by 75 and 78%, respectively, compared with the untreated cultures, while it reduced the growth of the wild-type strain and of the Δ*pchD* mutant by only 40 and 30%, respectively (Figure 3B). Altogether, these data indicate that the response of *P. aeruginosa* PAO1 to GaPPIX also depends on the carryover of intracellular iron.

### GaPPIX is preferentially uptaken *via* the *P. aeruginosa* PhuR receptor

**Figure 4:**
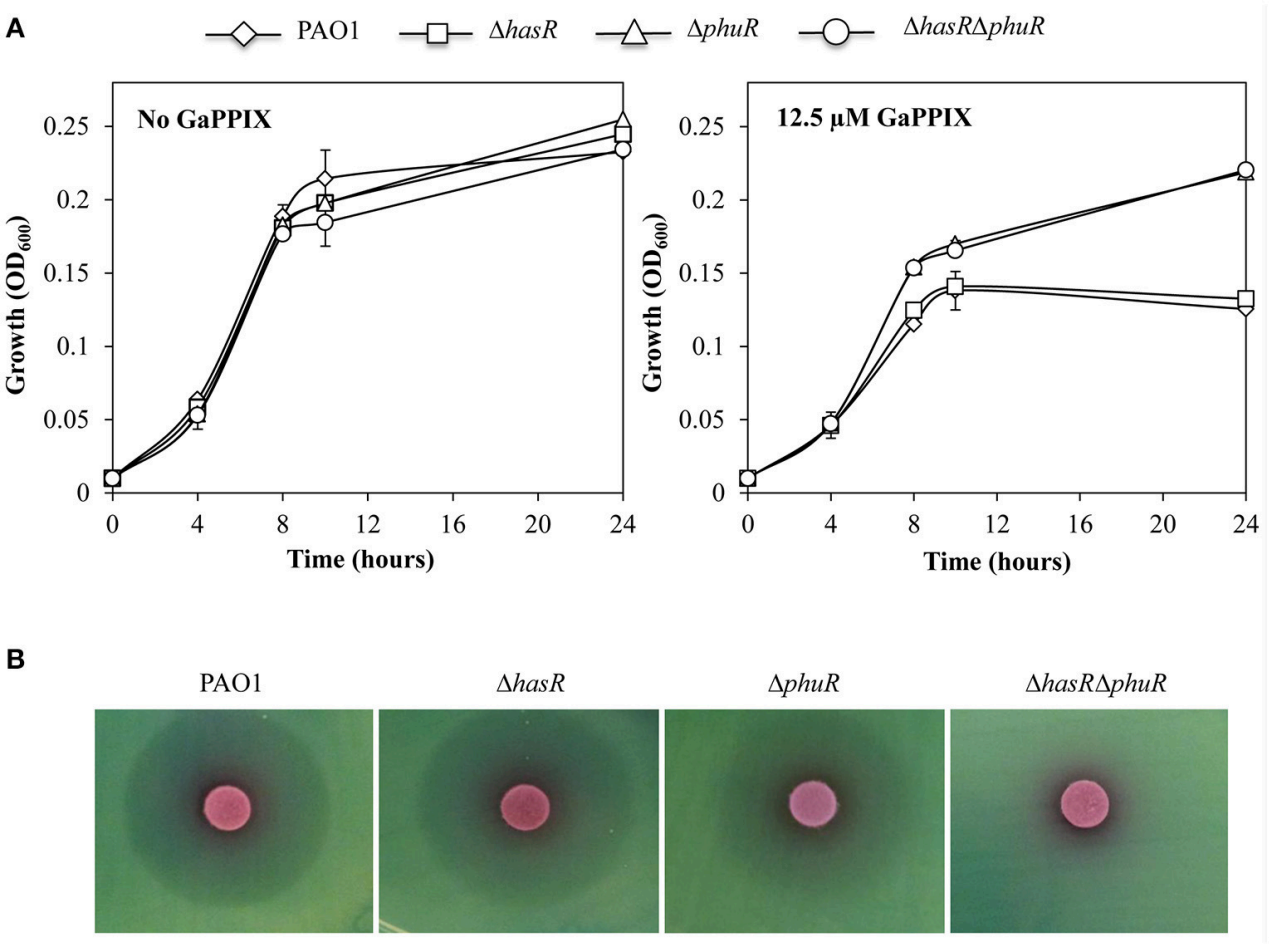
**GaPPIX enters ***P. aeruginosa*** cells through the heme-uptake systems. (A)** Growth of *P. aeruginosa* wild type (diamond) and Δ*hasR* (square), Δ*phuR* (triangle), and Δ*hasR*Δ*phuR* (circle) mutant strains in DCAA supplemented or not with 12.5 μM GaPPIX at 37°C. Values are the mean of two independent experiments, each one performed in duplicate ± the standard deviation. **(B)** Approximately 5 × 10^6^ bacterial cells were seeded on DCAA agar plates, then disks soaked with 10 μl of a 15 mM solution of GaPPIX were deposited on the agar surface. Plates were incubated for 16 h at 37°C. Images are representative of three independent experiments yielding similar results.

To investigate the hypothesis that GaPPIX may enter *P. aeruginosa* cells by exploiting the same routes as heme, *P. aeruginosa* mutants carrying a deletion of either of the known heme receptors (Δ*hasR* and Δ*phuR* mutants; Table 1) were generated. The effect of GaPPIX on these mutants, as well as on a Δ*hasR*Δ*phuR* double mutant lacking both heme receptors (Minandri et al., 2016), was investigated in DCAA in the presence of 12.5 μM GaPPIX (IC_50_; Figure 4A). While all strains showed the same growth profiles in the untreated medium, both Δ*phuR* and Δ*hasR*Δ*phuR* mutants grew better than the wild type or the Δ*hasR* mutant in the presence of 12.5 μM GaPPIX, displaying ≈50% higher growth levels relative to the wild type or the Δ*hasR* mutant (Figure 4A). These data suggest that, among the *P. aeruginosa* heme-uptake systems, *phu* has a more prominent role than *has* in the uptake of GaPPIX. Then, the effect of GaPPIX on heme-receptor mutants was evaluated in DCAA agar plates, by performing the disk diffusion assays (Figure 4B). Results showed a similar ZOI (27.6 ± 2.0 mm) for both the wild-type strain and the Δ*hasR* mutant, while a smaller ZOI (24.5 ± 0.7 mm) was observed for the Δ*phuR* mutant, indicating a less susceptible phenotype (Figure 4B, Table S2). In addition, no ZOI was observed for the Δ*hasR*Δ*phuR* double mutant, indicating a fully resistant phenotype (Figure 4B). These observations indicate that both *has* and *phu* systems are implicated in GaPPIX transport, although the *phu* system appears to be the preferential route for the entrance of GaPPIX in *P. aeruginosa* cells (Figure 4B).

### The sensitivity of *P. aeruginosa* to GaPPIX depends on the expression of the heme-uptake receptors

**Figure 5:**
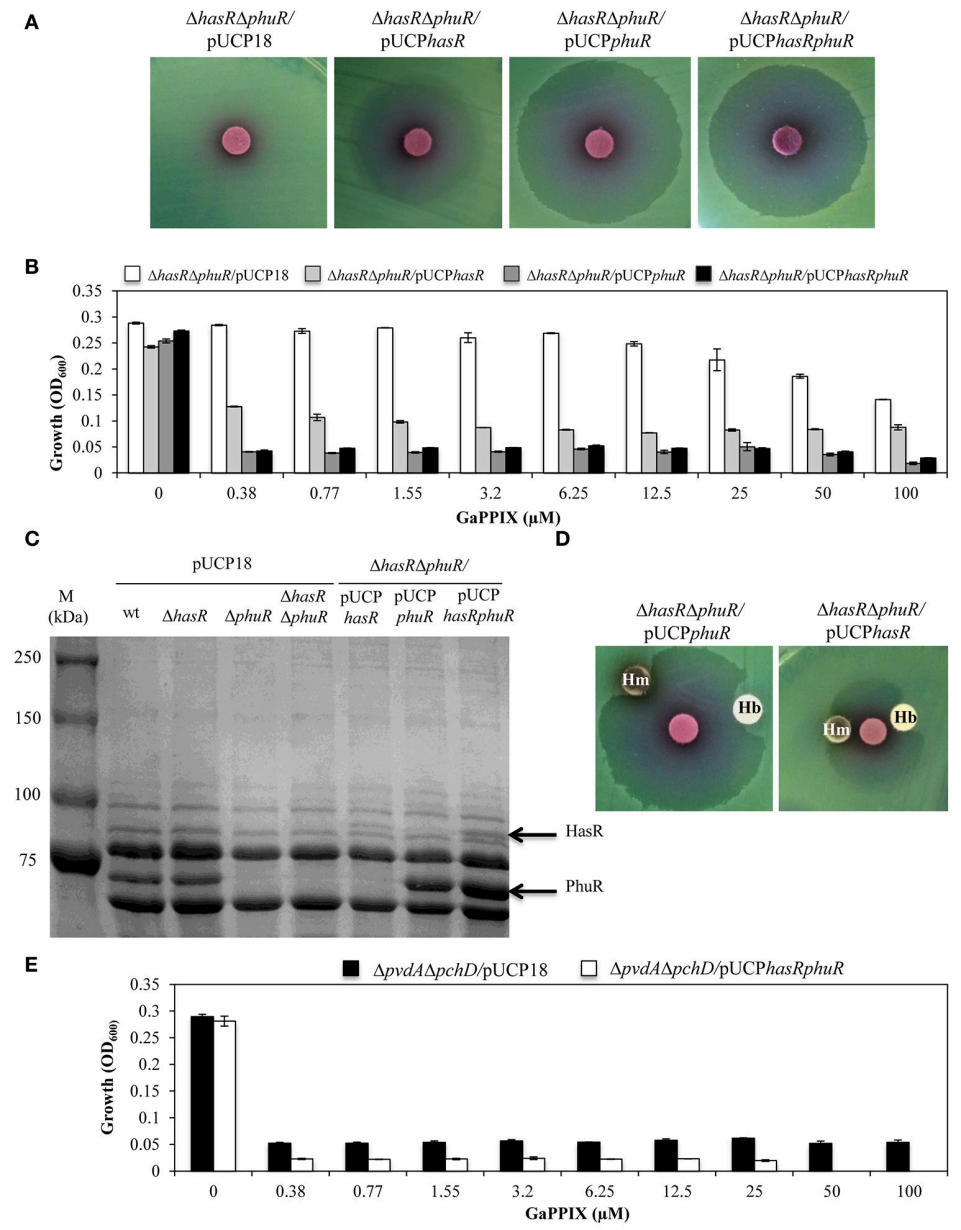
**The sensitivity of ***P. aeruginosa*** to GaPPIX depends on the expression of heme-uptake systems. (A)** Approximately 5 × 10^6^ bacterial cells were seeded on DCAA agar plates containing 200 μg/ml Cb, then disks soaked with 10 μl of a 15 mM solution of GaPPIX were deposited on the agar surface. Plates were incubated for 16 h at 37°C. **(B)** Growth of the *P. aeruginosa* Δ*hasR*Δ*phuR* mutant strain carrying the empty vector pUCP18 (white bars), or overexpressing *hasR* from plasmid pUCP*hasR* (light gray bars) or *phuR* from plasmid pUCP*phuR* (dark gray bars), or both *hasR* and *phuR* from plasmid pUCP*hasRphuR* (black bars), in DCAA supplemented with increasing concentrations of GaPPIX for 24 h at 37°C. **(C)** SDS-PAGE analysis of the outer membrane proteins of different *P. aeruginosa* PAO1 strains. Strains and plasmids are indicated on the top of each lane. M is the molecular mass marker (kDa), with band sizes on the left. The position of the putative 82 kDa PhuR and 94 kDa HasR outer membrane receptors is indicated by arrows on the right. **(D)** Rescue effect of hemin (Hm) or hemoglobin (Hb) from GaPPIX growth inhibition in the Δ*hasR*Δ*phuR* mutant strain overexpressing either *phu* from plasmid pUCP*phuR* or *hasR* from plasmid pUCP*hasR*. Approximately 5 × 10^6^ bacterial cells were seeded on DCAA agar plates containing 200 μM Cb. Blank discs were soaked with 10 μl of a 15 mM solution of GaPPIX or with 75 μg of either Hm or Hb. Plates were incubated at 37°C for 16 h. **(E)** Growth of the *P. aeruginosa* Δ*pvdA*Δ*pchD* mutant strain carrying the empty vector pUCP18 (black bars), or overexpressing both *hasR* and *phuR* from plasmid pUCP*hasRphuR* (white bars), in DCAA supplemented with increasing concentrations of GaPPIX for 24 h at 37°C. Values are the mean of two independent experiments, each one performed in triplicate ± the standard deviation. Images in **B** and **D** are representative of two independent experiments yielding similar results.

To further investigate the contribution of the HasR and PhuR receptors to GaPPIX-uptake, we individually expressed multicopy *hasR, phuR*, or both *hasR* and *phuR* in the Δ*hasR*Δ*phuR* mutant strain (using plasmids pUCP*hasR*, pUCP*phuR*, or pUCP*hasRphuR*, respectively) (Figure 5A). The effect of GaPPIX on these strains was initially tested by the disk diffusion assays (Figure 5A). While, the empty pUCP18 vector did not alter the susceptibility of Δ*hasR*Δ*phuR* to GaPPIX **(cfr** Figures 5A, 4B), the expression of *hasR* from the multicopy plasmid pUCP*hasR* made the Δ*hasR*Δ*phuR* mutant more susceptible to GaPPIX (ZOI = 27.6 ± 2.0 mm) (Figure 5A, Table S2). The effect of GaPPIX was even more pronounced in the Δ*has*Δ*phuR* mutant overexpressing either *phuR* (Δ*has*Δ*phuR* carrying the multicopy plasmid pUCP*phuR*; ZOI = 34.0 ± 1.0 mm) or both *hasR* and *phuR* (Δ*has*Δ*phuR* carrying the multicopy plasmid pUCP*hasRphuR*; ZOI = 33.3 ± 0.5 mm) (Figure 5A, Table S2). GaPPIX sensitivity of the Δ*has*Δ*phuR* strain expressing *hasR, phuR*, or both genes, was also evaluated in DCAA liquid medium, in the presence of different concentrations of GaPPIX (Figure 5B). All strains grew equally in the untreated medium, and GaPPIX did not affect the growth of Δ*hasR*Δ*phuR/*pUCP18 up to 25 μM (Figure 5B). Conversely, strains Δ*hasR*Δ*phuR/*pUCP*hasR*, Δ*hasR*Δ*phuR/*pUCP*phuR*, and Δ*hasR*Δ*phuR/*pUCP*hasRphuR* were very sensitive to GaPPIX. In particular, 0.38 μM GaPPIX reduced the growth of the Δ*hasR*Δ*phuR/*pUCP*hasR* strain by 56%, and by >80% in both Δ*hasR*Δ*phuR/*pUCP*phuR* and Δ*hasR*Δ*phuR/*pUCP*hasRphuR* strains (Figure 5B). This effect was much more pronounced than that observed for the parental strain PAO1 (Figure 2A). Of note, no further growth reduction was observed for both the Δ*hasR*Δ*phuR/*pUCP*phuR* and Δ*hasR*Δ*phuR/*pUCP*hasRphuR* mutant strains at > 0.38 μM GaPPIX. The increased sensitivity of the Δ*hasR*Δ*phuR* strain expressing either *hasR* or *phuR*, relative to the wild type, can be explained by the overexpression of heme receptors from the multicopy plasmid pUCP18 (Figure 5A). To confirm this hypothesis, HasR and PhuR protein levels were visualized by SDS-PAGE analysis of OMPs purified from the different *P. aeruginosa* strains cultured in DCAA (Figure 5C). By comparing *P. aeruginosa* outer-membrane-proteins profiles of the wild type, the Δ*phuR* or the Δ*hasR*Δ*phuR* mutant strains, the lack of a *ca*. 75 kDa protein in the Δ*phuR* or the Δ*hasR*Δ*phuR* mutants, was observed. This was in good agreement with a predicted molecular mass of 82 kDa for the mature PhuR receptor. Moreover, a protein band at that position was evident in SDS-PAGE electropherograms of the Δ*hasR*Δ*phuR/*pUCP*phuR* and the Δ*hasR*Δ*phuR/*pUCP*hasRphuR* complemented mutants (Figure 5C). Similarly, a protein band corresponding to *ca*. 94 kDa, consistent with the HasR receptor mass, was absent in the Δ*hasR* and Δ*hasR*Δ*phuR* mutants, while it was clearly detectable in the Δ*hasR*Δ*phuR/*pUCP*hasR* and Δ*hasR*Δ*phuR/*pUCP*hasRphuR* complemented mutants (Figure 5C). In line with previous results (Ochsner et al., 2000), protein levels greatly differed between PhuR and HasR, the latter being poorly expressed in wild-type PAO1. These results confirm that both HasR and PhuR direct GaPPIX entrance in *P. aeruginosa* cells, and argue for a prominent role of PhuR as a consequence of its higher expression levels, compared with HasR.

To confirm the specificity of GaPPIX for both heme-uptake systems, we investigated whether the growth inhibitory effect of GaPPIX could be rescued by the presence of Hemin (Hm) or Hemoglobin (Hb), which are known to deliver iron *via* heme-uptake receptors (Ochsner et al., 2000). To this aim, the heme-uptake mutant Δ*hasR*Δ*phuR* overexpressing either PhuR or HasR was tested in the GaPPIX disk diffusion assays in the presence of Hm and Hb (Figure 5D). Both Hm and Hb partly rescued the growth of the Δ*hasR*Δ*phuR* mutant overexpressing either PhuR (from pUCP*phuR*) or HasR (from pUCP*hasR*) thus confirming that (i) Hm, Hb and GaPPIX compete with heme receptors and (ii) GaPPIX enters *P. aeruginosa* cells through PhuR and HasR (Figure 5D).

It has been observed that *P. aeruginosa* isolates evolving during chronic lung infection in CF patients tend to accumulate mutations in siderophore loci, concomitant with preferential utilization of heme iron (Cornelis and Dingemans, 2013; Marvig et al., 2014; Andersen et al., 2015). To simulate this situation, we tested GaPPIX susceptibility of a siderphore-defective *P. aeruginosa* mutant overexpressing both PhuR and HasR receptors (Δ*pvdA*Δ*pchD/*pUCP*hasRphuR*). Whereas, exposure of the Δ*pvdA*Δ*pchD* mutant to GaPPIX reduced bacterial growth by 82% at 0.38 μM, expression of both *hasR* and *phuR* from multicopy plasmid pUCP*hasRphuR* made the Δ*pvdA*Δ*pchD* mutant extremely susceptible to GaPPIX, displaying 90% growth reduction (IC_90_) at 0.38 μM (Figure 5E). Notably, full inhibition of the Δ*pvdA*Δ*pchD*/pUCP*hasRphuR* strain was observed upon challenge with 50 μM GaPPIX.

### GaPPIX targets the aerobic respiration of *P. aeruginosa*

**Figure 6:**
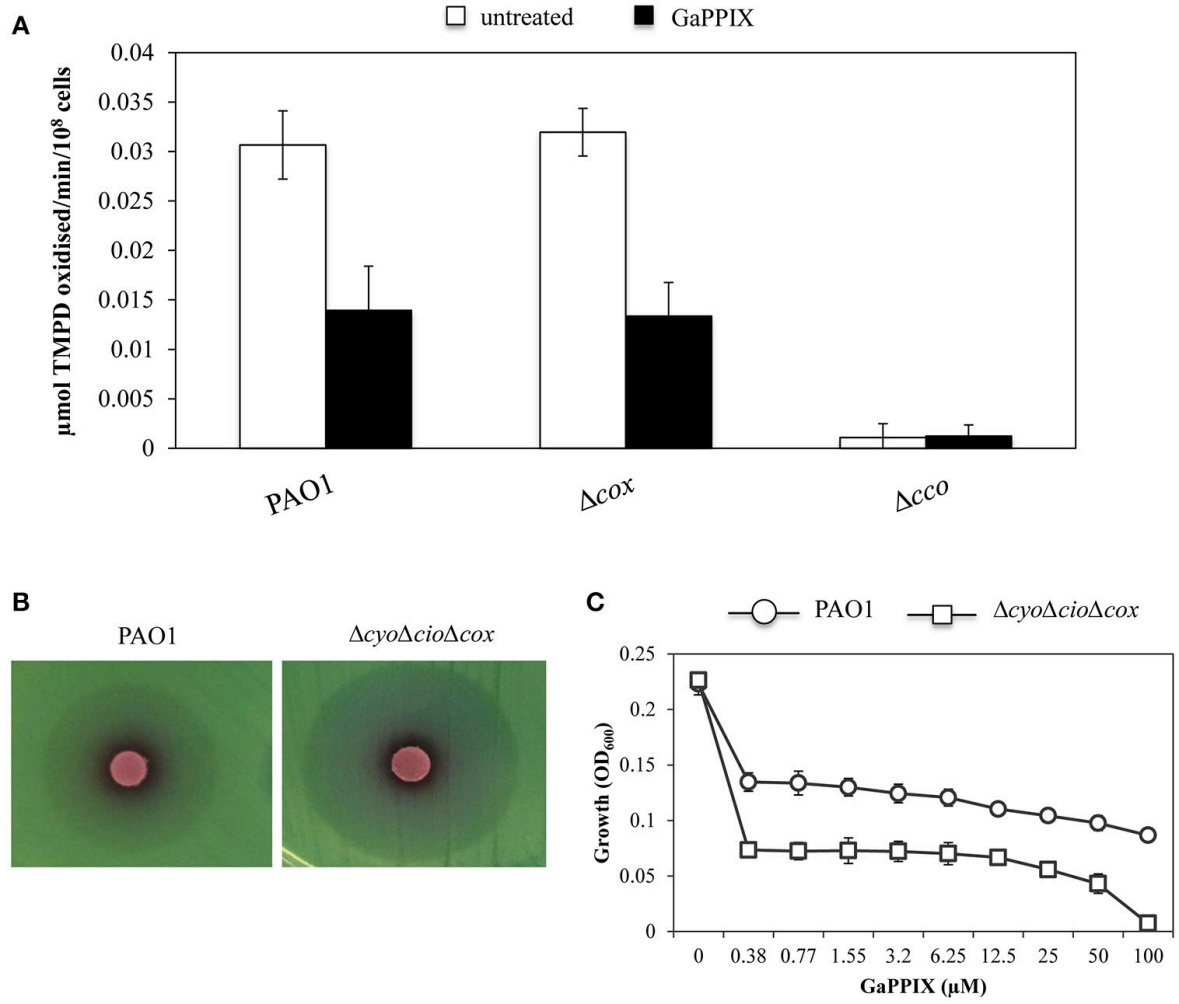
**GaPPIX inhibits ***P. aeruginosa*** PAO1 growth by targeting Cco. (A)** TMPD oxidase activity on whole cells of wild-type PAO1, Δ*cox* and Δ*cco* mutants grown for 6 h in DCAA supplemented (black bars) or not (white bars) with 4 μM GaPPIX. Activity is expressed as μmol TMPD oxidized/min^−1^/10^8^ cells at pH 7.0 and 25°C. Each value is the average of three independent experiments, each one performed in triplicate ± the standard deviation. **(B)** Approximately 5 × 10^6^ bacterial cells of wild-type PAO1 and the Δ*cyo*Δ*cio*Δ*cox* triple mutant were seeded on DCAA agar plates, then disks soaked with 10 μl of a 15 mM solution of GaPPIX were deposited on the agar surface. Plates were incubated for 16 h at 37°C. Images are representative of two independent experiments giving similar results. **(C)** Growth of wild type PAO1 (open circle) and the Δ*cyo*Δ*cio*Δ*cox* triple mutant (open square) in DCAA supplemented with increasing GaPPIX concentrations, for 24 h at 37°C. Values are representative of two independent experiments, each one performed in triplicate ± the standard deviation.

GaPPIX has been proven effective against a wide range of pathogenic bacteria by targeting metabolic pathways that require heme as an enzymatic cofactor, such as cellular respiration (Stojiljkovic et al., 1999). Thus, we investigated whether GaPPIX could interfere with the activity of terminal oxidases implicated in *P. aeruginosa* aerobic respiration. In particular, we focused on Cco-1, Cco-2, and Cio, which have been shown to sustain *P. aeruginosa* growth under low oxygen conditions, as those encountered in the lung of CF patients (Alvarez-Ortega and Harwood, 2007; Kawakami et al., 2010). To this aim, we initially tested the sensitivity of cytochrome *c* oxidases (i.e., Cox, Cco-1, and Cco-2) to GaPPIX. Strains deleted of the whole operon encoding the terminal oxidase Cox (Δ*cox*) or both the Cco-1 and Cco-2 terminal oxidases (Δ*cco*) were generated in the same parental strain used to generate the heme-receptor mutants (Table 1). The effect of GaPPIX was then assayed on these cytochrome-defective mutants using the TMPD redox indicator, which is an artificial electron donor to the cytochrome *c* (Matsushita et al., 1982). Oxidation of TMPD to a blue indophenol compound indicates electron flow to the cytochrome *c* terminal oxidases. Thus, cytochrome *c* oxidase activity was measured on *P. aeruginosa* PAO1 and in the Δ*cox* and Δ*cco* mutants grown in DCAA supplemented or not with a sub-inhibitory concentration of GaPPIX (4 μM). In whole cells cultured in the untreated medium, no cytochrome *c* oxidase activity could be measured in the Δ*cco* strain (Figure 6A), confirming that in our conditions the TMPD test mainly measures the activity of Cco. Indeed, the Δ*cox* mutation does not affect the TMPD oxidase activity (Figure 6A), as previously reported (Frangipani and Haas, 2009). This is because Cox is known to be poorly expressed during *P. aeruginosa* exponential growth (Kawakami et al., 2010). Interestingly, 4 μM GaPPIX reduced the respiratory activity by more than 50% in the wild-type strain PAO1 and the Δ*cox* mutant, compared with the untreated condition (Figure 6A). These observations suggest that Cco-1 and Cco-2 terminal oxidases are sensitive to GaPPIX. To confirm these preliminary results, the effect of GaPPIX was tested on a mutant expressing only Cco-1 and Cco-2. To this aim, a Δ*cyo*Δ*cio*Δ*cox* triple mutant strain was generated. Disk diffusion assays showed that the Δ*cyo*Δ*cio*Δ*cox* mutant was more sensitive to GaPPIX than the wild type (ZOI = 34.6 ± 1.24 vs 27.6 ± 2.0 mm, respectively) (Figure 6B, Table S2). Similar results were obtained in DCAA liquid cultures. PAO1 wild type and the Δ*cyo*Δ*cio*Δ*cox* mutant showed a similar growth profile in the untreated medium (Figure 6C), whereas exposure to 0.38 μM GaPPIX reduced bacterial growth by 40% and 68%, respectively, relative to the untreated cultures (Figure 6C). Interestingly, it was possible to determine an IC_90_ at 82 μM for the Δ*cyo*Δ*cio*Δ*cox* mutant strain (Figure 6C). These results confirm that Cco-1 and Cco-2 are targeted by GaPPIX.

**Figure 7:**
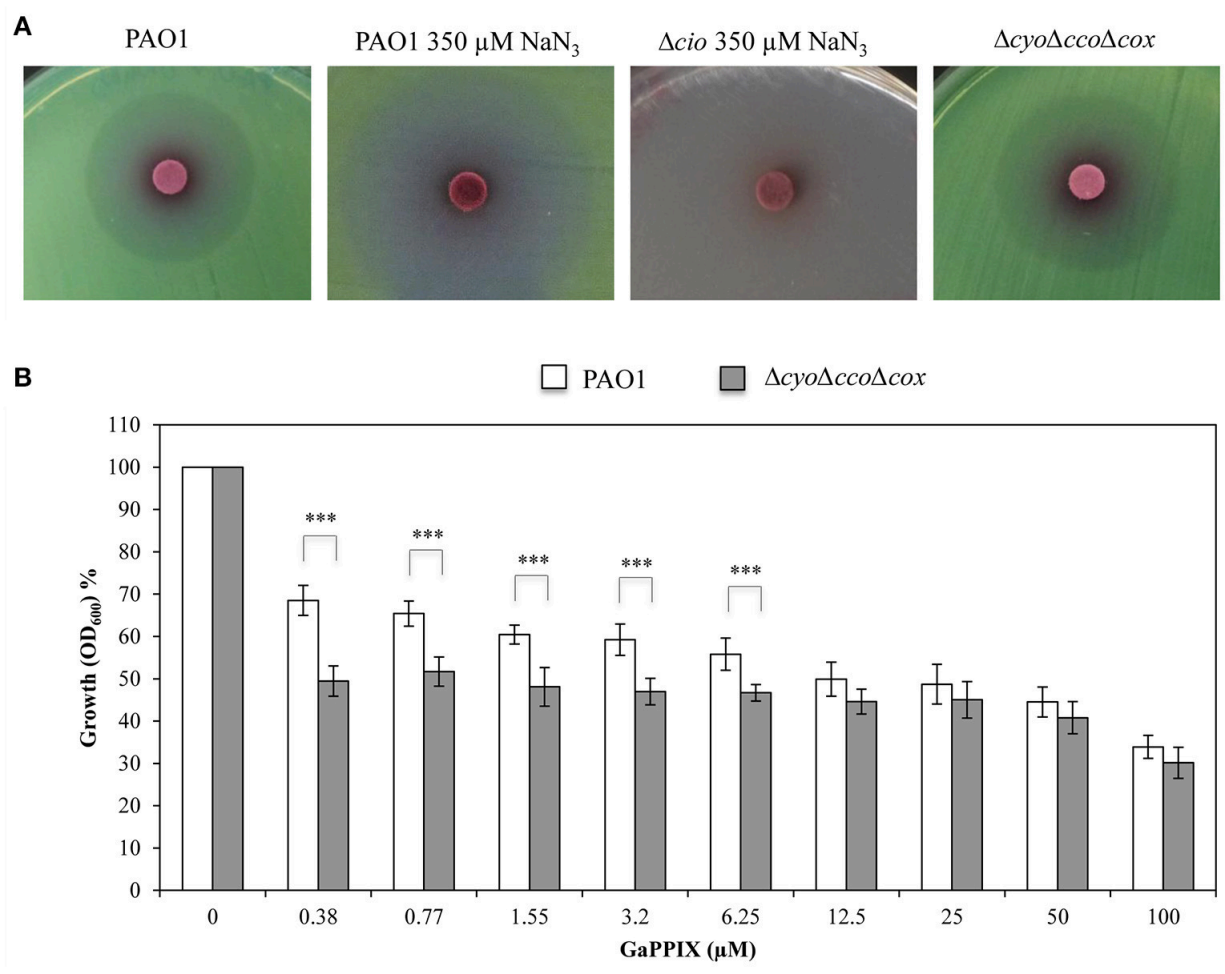
**GaPPIX inhibits ***P. aeruginosa*** PAO1 growth by targeting Cio. (A)** Approximately 5 × 10^6^ bacterial cells were seeded on DCAA agar plates, then disks soaked with 10 μl of a 15 mM solution of GaPPIX were deposited on the surface of DCAA agar plates supplemented or not with 350 μM NaN_3_. Plates were incubated for 16 h at 37°C. Strains and conditions are indicated on top of each panel. Images are representative of two independent experiments giving similar results. **(B)** Growth inhibition (%) of wild type PAO1 (white bars) and the Δ*cyo*Δ*cco*Δ*cox* triple mutant (gray bars) in DCAA supplemented with increasing concentrations of GaPPIX relative to the untreated controls (no GaPPIX). Growth was measured after 24 h incubation at 37°C. Values are representative of four independent experiments, each one performed at least in duplicate ± the standard deviation. Asterisks indicate statistically significant differences between the wild type PAO1 and the Δ*cyo*Δ*cco*Δ*cox* triple mutant (*P* < 0.001).

Then, the effect of GaPPIX on the Cio terminal oxidase was assessed. To this purpose, sodium azide (NaN_3_) was used as a specific inhibitor of copper-dependent oxidases, i.e., all terminal oxidases except Cio (Cunningham and Williams, 1995). Preliminarily, we determined the minimal NaN_3_ concentration inhibiting all terminal oxidases except Cio in DCAA, by comparing the growth of wild-type PAO1 and the Δ*cio* mutant in the presence of increasing NaN_3_ concentrations (250–1000 μM). We observed that 350 μM of NaN_3_ completely inhibited the Δ*cio* mutant without affecting PAO1 growth (data not shown). Then, the sensitivity of Cio to GaPPIX was tested by performing a GaPPIX disk diffusion assays with wild-type PAO1 in DCAA supplemented or not with 350 μM NaN_3_. It was observed that PAO1 remains sensitive to GaPPIX in the presence of 350 μM NaN_3_, displaying a ZOI even greater than that obtained for PAO1 without NaN_3_ (36.6 ± 3.0 vs 27.6 ± 2.0 mm, respectively) (Figure 7A, Table S2). This result provides evidence that Cio is a target for GaPPIX. To strengthen this evidence, a *P. aeruginosa* Δ*cyo*Δ*cco*Δ*cox* triple mutant, which expresses only Cio (Table 1) was constructed and assayed for GaPPIX susceptibility. Disk diffusion assay results showed that the Δ*cyo*Δ*cco*Δcox mutant was more sensitive to GaPPIX than the wild-type PAO1 (ZOI = 30.0 ± 0.7 vs 27.6 ± 2.0 mm, respectively) (Figure 7A, Table S2). Similar results were also obtained in DCAA liquid medium, showing that GaPPIX significantly reduced (*P* < 0.001) the growth of the Δ*cyo*Δ*cco*Δcox mutant relative to the wild type, at concentrations ranging between 0.38 and 6.25 μM (Figure 7B). Altogether, the above results indicate that *P. aeruginosa* Cco-1, Cco-2, and Cio terminal oxidases are targets for GaPPIX.

### *P. aeruginosa* clinical isolates are sensitive to GaPPIX

**Figure 8:**
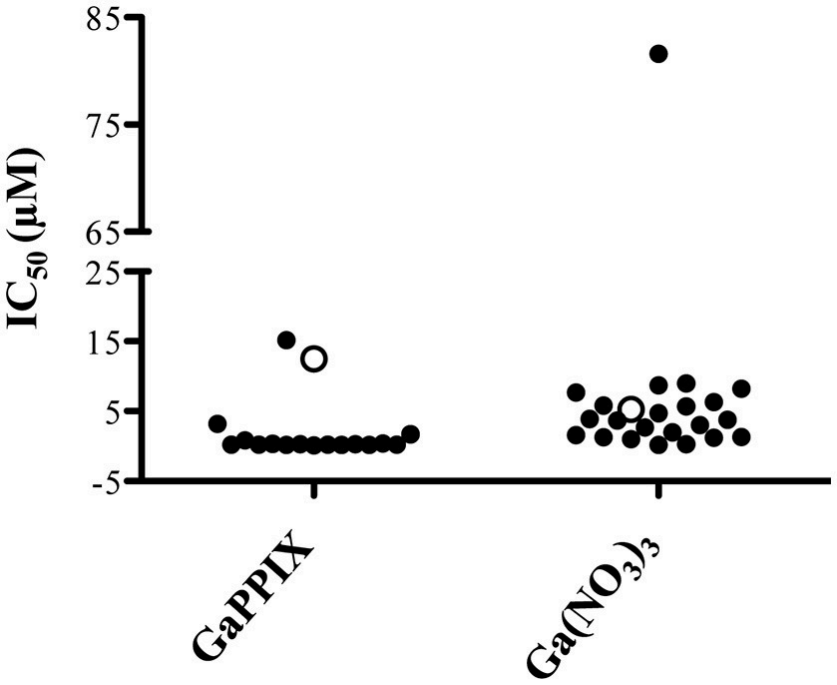
**Susceptibility of ***P. aeruginosa*** clinical isolates to GaPPIX**. IC_50_ values of GaPPIX or Ga(NO_3_)_3_ in a collection of *P. aeruginosa* clinical isolates (black) and in the reference strains PAO1 (white). Bacteria were grown for 24 h at 37°C in DCAA with increasing concentrations of GaPPIX or Ga(NO_3_)_3_ to determine the IC_50_. The IC_50_ of PAO1 was 12.5 μM for GaPPIX and 5.2 μM for Ga(NO_3_)_3_.

The expression of *P. aeruginosa* genes encoding heme-uptake systems has recently been detected in sputum samples collected from CF patients (Konings et al., 2013), and an evolution toward preferential heme utilization has been documented in *P. aeruginosa* during the course of chronic lung infection in CF patients (Marvig et al., 2014; Nguyen et al., 2014). Given the importance of heme in sustaining *P. aeruginosa* growth during infection, we have comparatively assessed the response to Ga(NO_3_)_3_ and GaPPIX in a collection of *P. aeruginosa* clinical isolates from CF and non-CF patients (Figure 8, Table S1). Although GaPPIX (up to 100 μM) never abolished *P. aeruginosa* growth, the majority of clinical isolates (>70%) was sensitive to GaPPIX, displaying an IC_50_ values in the range 0.1–15.2 μM (Table S1). Moreover, all but one *P. aeruginosa* clinical isolates were significantly more susceptible than the reference PAO1 strain (Figure 8). In line with previous reports (Bonchi et al., 2015), all clinical isolates except one (FM1, Table S1) were very sensitive to Ga(NO_3_)_3_, showing IC_50_ values ranging from 0.2 to 9 μM (Table S1).

## Discussion

The ability of pathogenic bacteria to colonize the host and cause infections is dependent on their capability to acquire iron and generate energy to sustain *in vivo* growth (Ratledge and Dover, 2000; Alvarez-Ortega and Harwood, 2007; Hammer et al., 2013). The success of *P. aeruginosa* as a pathogen relies on the presence of several iron-uptake systems (reviewed in Llamas et al., 2014), as well as on a multiplicity of terminal oxidases which allow bacterial respiration *in vivo*. Both iron-uptake systems and respiratory cytochromes have been shown to contribute to *P. aeruginosa* fitness during chronic lung infection in CF patients (Alvarez-Ortega and Harwood, 2007; Konings et al., 2013). Recent observations have documented an adaptation of *P. aeruginosa* toward heme iron acquisition in the CF lung, where bacterial energy metabolism mainly relies on the three terminal oxidases Cco-1, Cco-2, and Cio, all of which have high affinity for oxygen (Alvarez-Ortega and Harwood, 2007). These data suggest that heme utilization pathways and respiratory cytochromes could represent candidate targets for the development of new anti-*Pseudomonas* drugs (Alvarez-Ortega and Harwood, 2007; Marvig et al., 2014; Nguyen et al., 2014). Indeed, targeting bacterial membrane functions such as cellular respiration, are considered promising therapeutic opportunities, especially in the case of persistent or chronic infections (Hurdle et al., 2011). Given that all terminal oxidases require heme as a cofactor, and that heme-uptake systems are expressed during chronic lung infection, in this work we have investigated the effect of the heme-mimetic GaPPIX against *P. aeruginosa*. We focused on Cco-1, Cco-2, and Cio since *P. aeruginosa* uses any of these three terminal oxidases to support the microaerobic growth necessary to thrive in the lung of CF patients. Cox and Cyo are not expressed or strongly repressed under these conditions (Alvarez-Ortega and Harwood, 2007).

We have initially demonstrated that GaPPIX is able to reduce the growth of *P. aeruginosa* only under iron-limiting growth conditions. However, different from Ga(NO_3_)_3_, bacterial growth was never completely inhibited at GaPPIX concentrations up to 100 μM (Figure 2A), in line with the fact that the ZOI for GaPPIX was less transparent compared with that generated by Ga(NO_3_)_3_ in the disk diffusion assays (Figure 2B). This diverse response of *P. aeruginosa* upon exposure to GaPPIX or Ga(NO_3_)_3_ (Figures 2A,B) could be explained by the fact that GaPPIX and Ga(NO_3_)_3_ enter bacterial cells through different pathways. Ga(NO_3_)_3_ may enter *P. aeruginosa* cells (i) by diffusion; (ii) through the HitAB iron transport proteins (García-Contreras et al., 2013); or (iii) *via* the siderophore Pch (Frangipani et al., 2014). On the other hand, we have demonstrated that GaPPIX can cross the *P. aeruginosa* outer membrane only through the heme-receptors HasR and PhuR, since a Δ*hasR*Δ*phuR* mutant is fully resistant to GaPPIX (Figure 4). Indeed, overexpression of heme receptors in the Δ*hasR*Δ*phuR* mutant makes this strain susceptible to GaPPIX, at even lower GaPPIX concentrations compared with wild-type PAO1 (Figures 5A,B). However, it should also be taken into consideration that GaPPIX and Ga(NO_3_)_3_ likely have different targets. In fact, while Ga(NO_3_)_3_ is known to target a variety of essential iron-containing enzymes (Bernstein, 1998; Soo et al., 2016), less is known about GaPPIX targets. Several studies have demonstrated that the antibacterial activity of GaPPIX relies on the molecule as a whole, since GaPPIX cannot be cleaved by bacterial enzymes (Stojiljkovic et al., 1999; Hammer et al., 2013). In fact, we demonstrated that the homolog of GaPPIX (Hemin) did not affect *P. aeruginosa* PAO1 growth. Indeed, Hemin promoted bacterial growth at concentrations ranging between 1.55 and 25 μM (Figure 2A), likely as a consequence of iron delivery to the cell, combined with positive regulation of the *has* system (Llamas et al., 2014). Hence, GaPPIX might be erroneously incorporated in heme-containing proteins such as cytochromes. However, due to the multiplicity of pathways involving cytochromes, exposure to GaPPIX never results in a complete growth inhibition. This hypothesis is supported by the observation that GaPPIX is more active against *P. aeruginosa* mutants deleted in some of the cytochrome-dependent terminal oxidases (Figures 6, 7). In fact, a *P. aeruginosa* mutant that only expresses the terminal oxidases Cco-1 and Cco-2 (Δ*cyo*Δ*cio*Δ*cox*) is much more sensitive to GaPPIX than the wild-type strain. In addition, the Δ*cyo*Δ*cio*Δ*cox* mutant showed a 68% growth reduction in liquid DCAA at 0.38 μM GaPPIX, compared to the untreated cultures, and an IC_90_ of 82 μM (Figures 6B,C). Along the same lines, a *P. aeruginosa* strain that only relies on the terminal oxidase Cio to respire oxygen, is more sensitive to GaPPIX than the wild-type strain (Figure 7). Taken together, our results demonstrate that GaPPIX targets *P. aeruginosa* respiratory cytochromes Cco-1, Cco-2, and Cio, which are exclusively found in bacteria (Cunningham and Williams, 1995; Pitcher and Watmough, 2004), although we cannot discriminate which of the Cco cytochromes is preferentially targeted by GaPPIX (the Δ*cco*-1,2 strain is mutated in both). Moreover, it is tempting to speculate that GaPPIX may also inhibit the other terminal oxidases Cyo and Cox (Figure 1), as well as some of the enzymes involved in denitrification, such as the heme-containing protein complexes Nir and Nor (Figure 1). Moreover, GaPPIX could also be incorporated into heme-containing enzymes involved in the protection from oxidative stress, increasing the susceptibility of *P. aeruginosa* to reactive oxygen species.

Although it was not possible to determine the MIC of GaPPIX for wild-type PAO1, it is worth to point out that GaPPIX was extremely active against a *P. aeruginosa* mutant impaired in siderophore production (Δ*pvdA*Δ*pchD*) and overexpressing both HasR and PhuR heme receptors from plasmid pUCP*hasRphuR* (Figure 5). Ninety percent growth reduction and full inhibition were observed upon exposure of this mutant to 0.38 and 50.0 μM GaPPIX, respectively. It is tempting to speculate that such strong inhibition could also occur in the CF lung, where siderophore-defective *P. aeruginosa* variants emerge during chronic infection, and heme represents the principal iron source (Marvig et al., 2014; Nguyen et al., 2014). Inhibition could further be enhanced under the microaerobic conditions encountered by *P. aeruginosa* in the CF airways (Hogardt and Heesemann, 2010), where the three high affinity terminal oxidases targeted by GaPPIX (Cco-1, Cco-2, and Cio) are essential for bacterial growth (Alvarez-Ortega and Harwood, 2007). Irrespective of the Ga(III) delivery system and of the energy metabolism adopted by *P. aeruginosa*, the balance between Fe(III) and Ga(III) availability *in vivo* will be the main determinant of Ga(III) efficacy. The inhibitory activity of GaPPIX was not limited to the prototypic strain PAO1, as it was also exerted on a representative collection of *P. aeruginosa* clinical isolates (Table S1). The great majority of clinical isolates (>70%) was sensitive to GaPPIX, irrespective of their origin, and all but one were significantly more susceptible than PAO1 (IC_50_ ≤ 3.2 μM, Table S1).

Interestingly, studies on several human cell lines report that GaPPIX does not show cytotoxicity at concentrations ≤ 128 μM (Stojiljkovic et al., 1999; Chang et al., 2016), far above the concentrations that we found active on *P. aeruginosa* clinical isolates. Moreover, GaPPIX did not show to affect the health and behavior of mice, when administered by intraperitoneal injections (25–30 mg/kg) followed by four daily doses of (10–12 mg/kg) (Stojiljkovic et al., 1999), though it reduced the survival of *Galleria mellonella* larvae by 50% (LC_50_) when injected at 25 mM (Arivett et al., 2015).

Although further studies are needed to assess the effect of GaPPIX against *P. aeruginosa* infection *in vivo*, our work should encourage future research directed to the development of heme-mimetic drugs targeting cellular respiration for the treatment of *P. aeruginosa* chronic lung infection.

## Author contributions

PV and EF designed research; SH performed research; SH, EF, and PV analyzed data; SH, EF, and PV wrote the paper.

### Conflict of interest statement

The authors declare that the research was conducted in the absence of any commercial or financial relationships that could be construed as a potential conflict of interest.
