# Supplementary material for: Gallium-Protoporphyrin IX Inhibits Pseudomonas aeruginosa Growth by Targeting Cytochromes
Source: Front Cell Infect Microbiol. 2017 Jan 26;7:12. doi: 10.3389/fcimb.2017.00012 (PMC5266731; doi:10.3389/fcimb.2017.00012)
Supplement: Supplementary file 1 [file Table1.PDF]

Supplementary Material

Gallium-protoporphyrin IX inhibits *Pseudomonas aeruginosa* growth by targeting cytochromes

Sarah Hijazi[1], Paolo Visca[1] and Emanuela Frangipani[1]

[1]*Department of Science, Roma Tre University, Rome, Italy*

Correspondence: Emanuela Frangipani,
Department of Science,
Roma Tre University
Viale G. Marconi 446
00146 Roma, Italy
emanuela.frangipani@uniroma3.it

Table S1. *P. aeruginosa* clinical isolates used in this study.

**Strain**	**Origin**	**Source**	**GaPPIX IC_{50} (μM)**	**$Ga(NO_3)_3$ IC_{50} (μM)**	**Reference or source**
TB73	CF	rs	-	9	Bragonzi *et al.*, 2009
KK2	CF	rs	3.2	2.7	Bragonzi *et al.*, 2009
KK27	CF	rs	-	3.9	Bragonzi *et al.*, 2009
TR1	CF	rs	0.27	3	Bragonzi *et al.*, 2009
AA11	CF	rs	-	5.7	Bragonzi *et al.*, 2009
AA44	CF	rs	0.25	1.2	Massai *et al.*, 2011
FM1	CF	rs	0.32	81.6	Massai *et al.*, 2011
FM2	CF	rs	0.26	1.3	Massai *et al.*, 2011
FM4	CF	rs	0.2	1.6	Massai *et al.*, 2011
FM13	CF	rs	1.7	6.3	Massai *et al.*, 2011
FM17	CF	rs	0.38	4.7	Massai *et al.*, 2011
SP1	non-CF	ws	-	8.7	Bonchi *et al.*, 2015
SP6	non-CF	blood	15.2	3.7	Bonchi *et al.*, 2015
SP9	non-CF	cvc	-	5.8	Bonchi *et al.*, 2015
SP10	non-CF	blood	0.2	0.3	Bonchi *et al.*, 2015
SP11	non-CF	ascites	0.83	8.2	Bonchi *et al.*, 2015
SP15	non-CF	pe	0.21	0.2	Bonchi *et al.*, 2015
SP18	non-CF	bile	0.25	1.96	Bonchi *et al.*, 2015
SP20	non-CF	blood	0.19	1	Bonchi *et al.*, 2015
SP21	non-CF	pe	0.22	1.26	Bonchi *et al.*, 2015
SP24	non-CF	bile	-	7.7	Bonchi *et al.*, 2015
PA14	non-CF	burn	0.1	3.8	Rahme *et al.*, 1995
PAO1	non-CF	ATCC15692	12.5	5.25	American type culture collection

Abbreviations: CF, cystic fibrosis; rs, respiratory secretions; ws, wound swab; cvc, central venous catheter; pe, pleural exudate; - resistant.

Table S2. Effect of GaPPIX on different *P. aeruginosa* strains determined by mean of disk diffusion assays.

**Strain**	**GaPPIX susceptibility (avg ZOI ± SD)[a]**
PAO1	27.6 ± 2.0
Δ*hasR*	27.6 ± 2.0
Δ*phuR*	24.5 ± 0.7
Δ*hasR*Δ*phuR/*pUCP*hasR*	27.6 ± 2.0
Δ*hasR*Δ*phuR/*pUCP*phuR*	34.0 ± 1.0
Δ*hasR*Δ*phuR/*pUCP*hasRphuR*	33.3 ± 0.5
Δ*cyo*Δ*cio*Δ*cox*	34.6 ± 1.24
PAO1 in the presence of 350 µM NaN_3	36.6 ± 3.0
Δ*cyo*Δ*cco*Δ*cox*	30.0 ± 0.7

[a] ZOIs were measured in mm; Average (avg) is the mean of at least three independent experiments.

Additional references not included in the main text:

Bragonzi, A., Paroni, M., Nonis, A., Cramer, N., Montanari, S., Rejman, J., Tümmler, B., et al. (2009). *Pseudomonas aeruginosa* microevolution during cystic fibrosis lung infection establishes clones with adapted virulence. *Am. J. Respir. Crit Care Med.* 180, 138-145. doi: 10.1164/rccm.200812-1943OC

Massai, F., Imperi, F., Quattrucci, S., Zennaro, E., Visca, P., Leoni, L., et al. (2011). A multitask biosensor for micro-volumetric detection of N-3-oxo-dodecanoyl-homoserine lactone quorum sensing signal. *Biosens. Bioelectron*. 26, 3444-3449. doi: dx.doi.org/10.1016/j.bios.2011.01.022

Rahme, L. G., Stevens, E. J., Wolfort, S. F., and Shao, J. (1995). Common virulence factors for bacterial pathogenicity in plants and animals. *Science* 268, 1899.
